# Abscopal Effect, From Myth to Reality: From Radiation Oncologists' Perspective

**DOI:** 10.7759/cureus.3860

**Published:** 2019-01-09

**Authors:** Melek Tugce Yilmaz, Aysenur Elmali, Gozde Yazici

**Affiliations:** 1 Radiation Oncology, Hacettepe University Medical School, Ankara, TUR

**Keywords:** abscopal effect, immunotherapy, radiotherapy

## Abstract

The abscopal effect is mediated by a systemic anti-tumor immune response and reflects the regression of non-irradiated metastatic lesions at a distance from the primary site of irradiation. This review will focus on understanding the biological rationale behind the abscopal effect of radiotherapy (RT), which has a recently renewed interest as a result of the successes achieved with immunotherapy and RT in combination. Both RT and immunotherapy are standard components of modern treatment regimens. Combination of these two modalities results in an increased response in the irradiated lesions themselves and the metastatic regions distant from the site of irradiation. We will summarize the abscopal effect of radiotherapy, in particular, the synergistic effect of RT and immunotherapy.

## Introduction and background

Radiotherapy (RT) is a treatment modality which is used to achieve local control of the irradiated area. However, there may be a tumor response in areas where RT has not been performed. This phenomenon is called the abscopal effect of radiotherapy. However, the biological mechanism underlying this effect is not yet clear.

The direct and indirect effects of radiation on deoxyribonucleic acid (DNA) are the primary mechanism responsible for the cytotoxic effects of radiotherapy. In addition to the direct cytotoxic effect on tumor cells, the impacts on the immune system have also been the subject of studies for so long. These effects on the immune system can result in an immune-mediated anti-tumor response. However, a limited number of cases with abscopal effect have been reported where RT was performed solely.

## Review

What is the abscopal effect and how can it be combined with immunotherapy?

The word “abscopal” was first used by Mole in 1953 [1]. Abscopal means "at a distance from the irradiated volume but within the same organism,” and is derived from ab-, prefix with the meaning "position away from" and scopus (Latin) meaning "mark or target for shooting at".

Recently, the cancer treatment approach has changed drastically, and the fight against cancer with immune-stimulation has opened a new era of immunotherapy. The treatment with immune checkpoint inhibitors made an old term, the abscopal effect, popular again in the radiation oncology community.

RT is one of the cornerstones of cancer treatment in definitive and the palliative settings with well established local effects. Classical radiobiology postulates that the cytotoxic effects of radiation are the result of direct DNA damage and indirect generation of cell-damaging free radicals [2]. This makes this modality precisely a local therapy which is characterized by an impressively high degree of spatial accuracy. Additional research has demonstrated that RT also causes localized bystander effects, where the treated tumor cells influence neighboring cells [3]. Because of all these cytotoxic effects and the effects on circulating lymphocytes RT is known to be immunosuppressant, but by the definition of the abscopal effect, there might be an immune stimulant nature of RT [1].

RT does not only exerts direct cytotoxic effects on tumor cells but also reprogrammes the tumor microenvironment to exert a potent antitumor immune response and enhances antitumor immunity [4]. It initiates immunogenic cell death causing production and release of the cytokines and chemokines into the tumor microenvironment. This causes chemoattraction and infiltration of dendritic cells (Dcs) to the site of the tumor. Activation of Dcs, which are essential antigen presenting cells, and up-regulation of cytotoxic T lymphocytes are responsible for the abscopal effect mechanism [4-7]. On the other hand, the infiltration of Tregs and myeloid-derived suppressor cells (MDSCs) act as a brake mechanism on the immune system.

As a part of the RT’s nature, which is immunosuppressive dominantly, abscopal is seen as a rare event. Over the years sporadic abscopal effect events were published as case reports. Over the years sporadic abscopal effect events were published as case reports. Abuodeh et al. reviewed the literature and reported that 46 case reports were published between 1969 and 2014 where the abscopal effect was observed from RT alone. When we look closely, in the reviewed cases, the abscopal effect was observed independently from the treatment dose, fractionation, modality and the characteristic of the target lesion. The dose range was 0,45 Gy to 74,8 Gy, and the abscopal effect was seen at the second week earliest. Moreover, most of the reported cases were on renal cell carcinoma, melanoma, and lymphoma, which are the disease entities traditionally considered immunogenic [8].

Due to the rarity of this phenomenon, many methods to increase the prevalence of the abscopal effect have been tried up to date. These strategies were based on the combination of RT with cytokines, stimulation of Dcs to aggravate tumor antigen-presenting, vaccination with autologous tumor cells, targeting Toll-like receptors and immunotherapy-RT combination [9-13]. Dewan et al. tried to find the optimal dose and fractionation schemes of RT to induce the abscopal effect with immunotherapy. They showed that fraction dose of RT might be a critical determinant of its efficacy to synergize with immunotherapy [14]. In their experimental design, 8 Gy given in three consecutive days (8Gyx3) was the most effective regimen for induction of the abscopal effect in combination with cytotoxic T-lymphocyte–associated antigen 4 (CTLA-4) blockade [14]. Later they studied the underlying mechanisms for this dose effect. They showed that the balance between the cytosolic DNA and DNA exonuclease Trex1 activation plays a vital role in the synergistic effect of RT and immunotherapy. RT induces double-strand DNA accumulation in the cytosol, and the amount of double-strand DNA is directly proportional to the fraction dose of RT. Cytosolic DNA has an essential impact on the activation of antitumor immunity, as it activates the DNA sensor cyclic GMP-AMP (cGAMP) synthase (cGAS) and its downstream effector, stimulator of interferon genes (STING). Activation of this cascade results in interferon-b secretion by cancer cells, which causes dendritic cell recruitment and activation. This stimulus is essential for priming of CD8 T cells and antitumor immunity. On the other hand, doses above 12–18 Gy per fraction cause activation of DNA exonuclease Trex1. Activated DNA exonucleases degrade cytosolic DNA and attenuate the immunologic response stimulated by RT. This delicate balance between cytosolic DNA and activated Trex1 is optimal at 8Gyx3 for the emergence of the !abscopal effect with immunotherapy [15].

**Figure 1:**
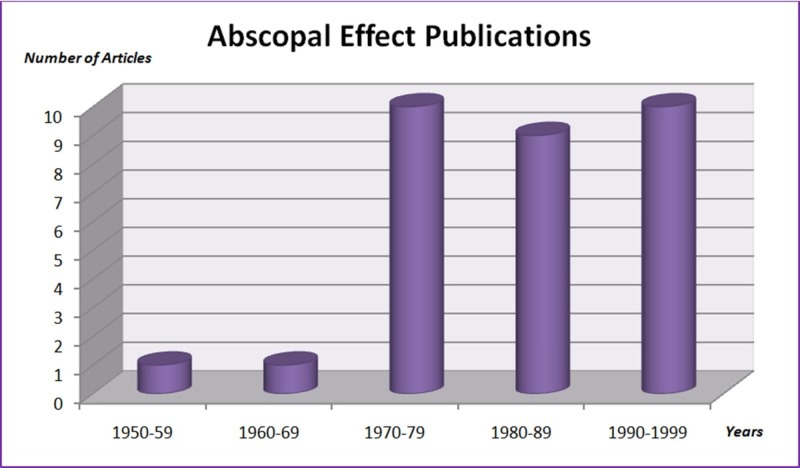
Abscopal effect publications from 1950 to 1999 "Abscopal effect" term was searched in Medline from 1950 to 1999. Each bar represents the number of articles in the corresponding ten years period.

**Figure 2:**
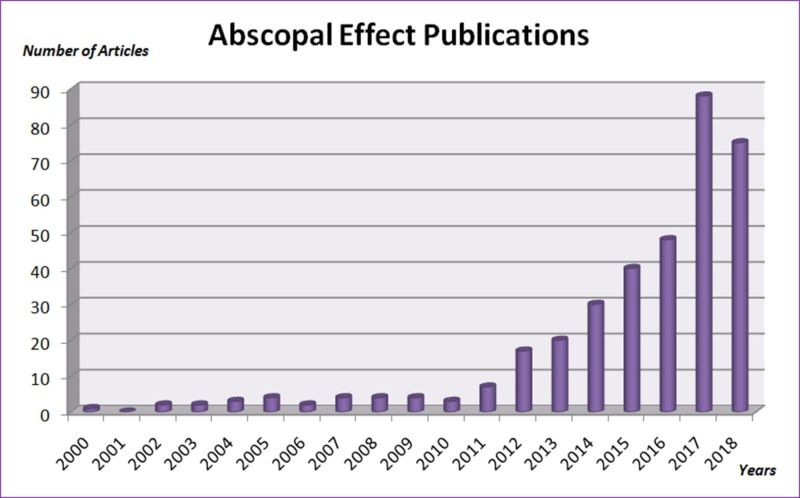
Abscopal effect publications from 2000 to date "Abscopal effect" term was searched in Medline from 2000 to date. Each bar represents the number of articles in that year.

With the new modern immunotherapy era, the potential for immune activation by radiation therapy defines an important role for radiation therapy in systemic disease. Therefore all of this brought us to the period of achieving the maximum abscopal effect by using immunotherapy and RT simultaneously. An increasing number of reports of abscopal effect is seen in cases where RT is combined with various immunotherapeutic agents that enhance the immune response or remove the inhibitory effect of existing immune checkpoints (Figures 1-2).

Postow et al. were the first to report an abscopal effect in a metastatic melanoma case treated with ipilimumab (a monoclonal antibody that inhibits an immune checkpoint on T cells, the cytotoxic T lymphocyte-associated antigen 4) and RT. The patient had progressive disease during maintenance treatment with ipilimumab. RT was delivered to a painful paraspinal mass with a palliative intent, which led to a systemic response [16]. Reynders et al. published an overview of the abscopal effect of RT in 2015. They inclueded studies that used immmune modifying therapy but excluded studies using total body irradiation and/or cytotoxic agents. They reviewed 26 clinical reports, 23 case reports, and one retrospective study. The median time to abscopal response was five months and was maintained for three to 39 months [17]. Even further, Poleszczuk and Enderling suggested a mathematical model that can be used to predict and dissect the complexity of the immune-mediated response at multiple tumor sites after applying focal irradiation and systemic immunotherapy [18].

In a recently published article by Yazici et al., the abscopal effect is demonstrated in a sinonasal carcinoma patient. Irradiation the target lesion with concurrently pembrolizumab resulted in resolution of all the metastatic lesions. In this patient, pembrolizumab was used as second-line therapy, and tumor progression was observed under pembrolizumab. When RT came into the picture and combined with immunotherapy, complete treatment response was achieved [19].

## Conclusions

In the light of scientific advances in human biology and understanding of the immune system as a result of evolving technology and medical advances, it is clear that immunotherapy may replace cytotoxic systemic therapies in the future. As a result of the synergistic effect of such promising immunotherapy with RT, a good tumor response can be achieved in both irradiated and distant metastases. Regardless of prospective or retrospective data, all data are valuable and promising for the future.
